# Single-step electrochemical functionalization of double-walled carbon nanotube (DWCNT) membranes and the demonstration of ionic rectification

**DOI:** 10.1186/1556-276X-8-279

**Published:** 2013-06-10

**Authors:** Xin Zhan, Ji Wu, Zhiqiang Chen, Bruce J Hinds

**Affiliations:** 1 Department of Chemistry, University of Kentucky, Lexington, KY 40506, USA; 2 Department of Chemical and Materials Engineering, University of Kentucky, Lexington, KY 40506, USA; 3 Current address: Department of Chemistry, Georgia Southern University, Statesboro, GA 30460, USA

**Keywords:** Carbon nanotube membranes, Rectification, Voltage gatekeeper, Amine electrooxidation

## Abstract

Carbon nanotube (CNT) membranes allow the mimicking of natural ion channels for applications in drug delivery and chemical separation. Double-walled carbon nanotube membranes were simply functionalized with dye in a single step instead of the previous two-step functionalization. Non-faradic electrochemical impedance spectra indicated that the functionalized gatekeeper by single-step modification can be actuated to mimic the protein channel under bias. This functional chemistry was proven by a highly efficient ion rectification, wherein the highest experimental rectification factor of ferricyanide was up to 14.4. One-step functionalization by electrooxidation of amine provides a simple and promising functionalization chemistry for the application of CNT membranes.

## Background

A protein channel embedded in a cell membrane functions as a natural regulator in the biological system. Conformational change of proteins actuated by voltage can open or close the gate of the channel, which regulates ion permeation with high selectivity [1-4]. It inspires researchers to develop artificial nanopores and nanochannels in response to external signals (voltage, pH, temperature, light, etc.) by mimicking natural ion channels [5]. Transmembrane voltage is an excellent stimulus to open or close the gate of a nanodevice since it is not aggressive, is tunable, and can act over a short time scale [6]. Therefore, it can modulate ionic flux and rectify ionic transport current through the nanochannel/nanopore. These nanodevices acting as rectifier enable the possible applications in single-molecule sensing and separation [7-10]. Carbon nanotube (CNT) membranes offer a fast fluid platform. The fluid velocity of a carbon nanotube membrane is 10,000 times faster than the conventional membrane of similar pore size due to atomically smooth graphite core [11,12]. Moreover, the CNT membranes have far more mechanical strength than lipid bilayer films, thus providing an exciting opportunity for chemical separation, drug delivery, and other applications [13,14]. Carbon nanotube membranes can imitate ion channels with functionalized molecules acting as mimetic gatekeepers. Chemical functionalization of molecules (biotin [15], phosphorylation [16], and charged dye [17]) at the entrance of the CNT core enables the modest modulation of ionic transportation. Further study had shown that the steric hindrance of gatekeepers at the pore entrance can be controlled with voltage [18]. Negative bias repels the anionic tethered molecules away from the CNT entrance, opening the channel, while positive bias pulls the anionic tethered molecules into the pore, thus closing the channel. The voltage-gated carbon nanotube membranes have been successfully applied in drug delivery. CNT membranes enable the programmable delivery of the addictive drug nicotine into the human skin *in vitro* for abuse treatment [19]. Neutral caffeine can also be pumped through CNT membranes via a highly efficient electroosmotic flow that is 100-fold more power efficient compared to conventional materials such as anodized aluminum oxide membranes [20].

To achieve gatekeeper activity on CNT membranes, there needs to be a high functional density only at the CNT tips or pore entrances [12,21]. This has been largely achieved with a two-step process, wherein diazonium grafting first creates carboxyl groups at the CNT tips followed by carbodiimide coupling chemistry [17,22]. Diazonium grafting generates highly reactive radicals that covalently react with the electrode or subsequent organic layer on the surface under mild solvent and temperature conditions [23,24]. However, it is difficult to control the amount of carboxylate groups on the CNT tip due to polymerization during diazonium grafting [24,25]. In principle, grafting reaction is self-limiting when an insulating polymer layer stops the electrochemical reduction of diazonium salt. However, with ionic functional groups (such as carboxylates), the reaction can proliferate and block carbon nanotubes. Another complication of the diazonium approach is that it generally requires two-step functionalization since the diazonium formation reaction is not compatible with many functional groups that would be required on the gatekeeper. This adds complication and reduces the overall yield. Electrochemical oxidation of amine to coat carbon fiber surface predates diazonium grafting with its first report in 1990 [26]. It enables immobilization of various primary amine-containing molecules on different electrode surfaces [27-31]. The electrografted layer is characterized by atomic force microscopy, X-ray photoelectron spectroscopy, ellipsometry, time-of-flight secondary ion mass spectrometry, and electrochemistry methods [32-34]. Amine electrochemical oxidation greatly simplifies the surface modification process since it does not need complicated synthesis and surface chemistry. Even large molecules including dendrimers and metal-ligand complex can be directly functionalized on a conductive surface in a single step [35-38]. Electrografting of amine offers a simple and efficient functional chemistry for CNT applications. Electrografting of amine provides binding sites on CNTs for the coating of Pt-Ru and Ag nanoparticles that exhibit excellent electrocatalytic activity [39,40]. The more controllable electrochemical grafting of the fluorinated aminobenzoic acid layer enables the Pt monolayer deposition on CNT buckypaper. The highest record of mass activity has been achieved at 2,711 A g^−1^ in methanol oxidation [41].

The primary hypothesis of this paper is that the efficiency of voltage gatekeeping can be enhanced to obtain high on/off ratio using electrooxidation of amine in one step. The conformational changes of tethered dye molecules under bias will be identified by non-faradic electrochemical impedance spectroscopy (EIS) measurements. The EIS spectra can prove the effectiveness of this single-step functionalization on double-walled carbon nanotube (DWCNT) membranes. Transmembrane ionic rectification will be measured to compare the efficiency of gatekeeping. Stronger rectification indicates more efficient gatekeeping. The gatekeeper density is still unknown in our previous work. This can be quantified by dye assay on glassy carbon due to its similar structure with CNTs. A single-step modification may give higher overall functional density over a complicated two-step modification.

## Methods

### Fabrication of double-walled carbon nanotube membranes

**Figure 1:**
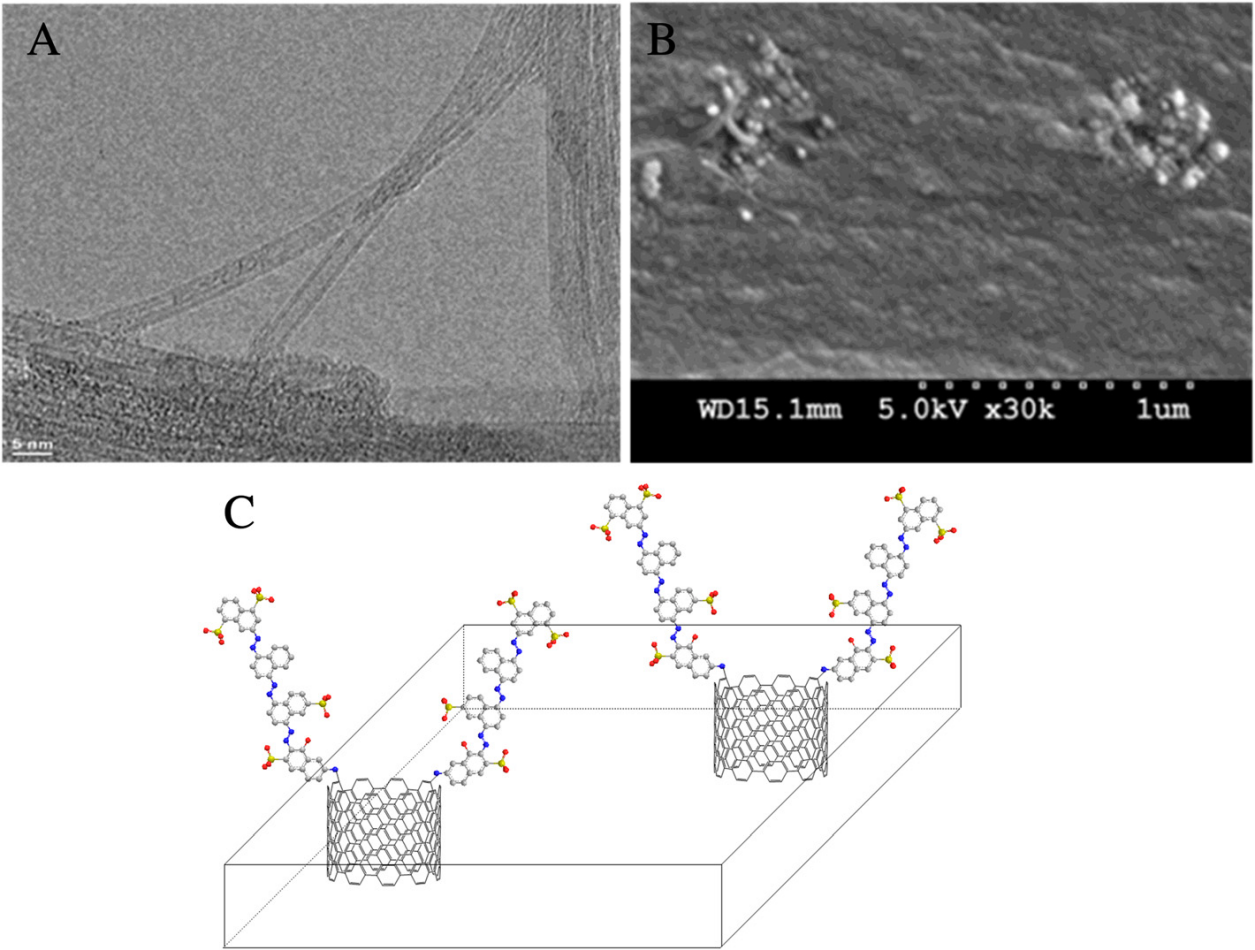
**TEM and SEM images of DWCNT and schematic diagram of functionalized anionic dye.** (**A**) TEM image of DWCNTs (purchased from Sigma-Aldrich). (**B**) SEM image of as-made DWCNT membrane in the cross-sectional view. (**C**) Schematic diagram of functionalized anionic dye on the CNT tip playing as gatekeeper (gray, C; red, O; blue, N; yellow, S).

DWCNTs with average inner diameter of 2 nm and length of 30 μm were purchased from Sigma-Aldrich Corporation (St. Louis, MO, USA; transmission electron microscopy (TEM) image as seen in Figure 1A). DWCNT membranes were fabricated using microtome cutting method similar to that in previous reports [19,20,42]. To describe it briefly, 5 wt.% CNTs were mixed with Epoxy 862 epoxy resin (Miller Stephenson Chem. Co., Danbury, CT, USA), hardener methylhexahydrophthalic anhydride (Broadview Technologies, Newark, NJ, USA), and 0.1 g surfactant Triton-X 100 (Sigma-Aldrich) using a Thinky™ (Tokyo, Japan) centrifugal shear mixer. As-prepared CNT-epoxy composite was cured at 85°C according to the commercial epoxy procedure before being cut into CNT membranes using a microtome equipped with a glass blade. The typical thickness of as-cut CNT membrane is 5 μm (Figure 1B). The membranes (approximately 0.6 × 0.6 cm^2^) were glued over a 3-mm diameter hole in polycarbonate plate (1-mm thick) to act as mechanical support. The top of the membrane was referring to the surface in the recess of the hole in the polycarbonate support, while the bottom of the membrane was on the bottom plane of the polycarbonate support. Pd/Au (30 nm) was sputter-deposited on the bottom of the membrane to give electrical contact to the CNT membrane and to act as effective working electrode.

### Modification of DWCNT membranes

To avoid grafting in the inner core of CNTs, CNT membranes were placed in U-tube fittings under a 2-cm inner solution column pressure. In two-step functionalization, as-prepared DWCNT membranes were first modified by flow electrochemical grafting with 5-mM 4-carboxy phenyl diazonium tetrafluoroborate/0.1-M KCl solution at −0.6 V for 2 min. In the next step, Direct Blue 71 dye (Sigma-Aldrich) was coupled with the carboxyl group on the tip of CNTs with carbodiimide chemistry: 10 mg of ethyl-(*N*′,*N*′-dimethylamino) propylcarbodiimide hydrochloride and 5 mg of *N*-hydroxysulfosuccinimide were dissolved into 4 ml of 50-mM Direct Blue 71 dye in 0.1 M 2-(*N*-morpholino) ethane sulfonic acid buffer for 12 h at ambient temperature.

In one-step functionalization, Direct Blue 71 dye, which has a primary amine, was directly grafted to CNT by electrooxidation of amine. Electrografting was carried out under a constant potential of 1.0 V using a potentiostat (E-corder 410, eDAQ, Denistone East, Australia) in the three-electrode cell. The CNT membrane, with sputtered Pd/Au film (approximately 30-nm thick) on the membrane's back side, was used as the working electrode; Pt wire was the counter electrode, and the reference electrode was Ag/AgCl. Before electrografting, the ethanol solution of 0.1 M LiClO_4_/1 mM direct blue was purged by argon gas for 15 min to remove adsorbed oxygen in the solution.

### Rectification experimental setup

The schematic of the ionic rectification setup is shown in Additional file 1: Figure S1. Both U-tube sides were filled with potassium ferricyanide solution. The working electrode (W.E) was DWCNT membrane coated with 30-nm-thick Pd/Au film; the reference electrode (R.E) was Ag/AgCl electrode. Voltage was controlled using an E-Corder 410 potentiostat. The counter electrode was a sintered Ag/AgCl electrode purchased from IVM Company (Healdsburg, CA, USA). The membrane area was approximately 0.07 cm^2^. Linear scan was from −0.60 to +0.60 V with the scan rate at 50 mV/s.

### Dye assay quantification of carboxyl and sulfonate density on glassy carbon

Toluidine blue O was reported to quantify the carboxyl group density on the polymer film. Our dye assay method was similar to that of previous reports [43,44]. Glassy carbon was incubated in 0.2-mM toluidine blue O (TBO, Sigma-Aldrich) solution at pH 10 and at room temperature for 1 h to adsorb positively charged dye onto the anionic carboxylate or sulfonate group. The glassy carbon was then rinsed with NaOH (pH 10) solution and further incubated in 0.1-mM NaOH (pH 10) solution for 5 min to remove physisorbed TBO dye. The adsorbed TBO on anionic glassy carbon was removed from the HCl solution (pH 1). The concentration of desorbed TBO in the HCl solution was determined by the absorbance at 632 nm using Ocean Optics (Dunedin, FL, USA) USB 4000 UV–vis spectrometer. The calculation of carboxyl or sulfonate density was based on the assumption that positively charged TBO binds with carboxylate or sulfonate groups at 1:1 ratio on glassy carbon.

## Results and discussion

**Figure 2:**
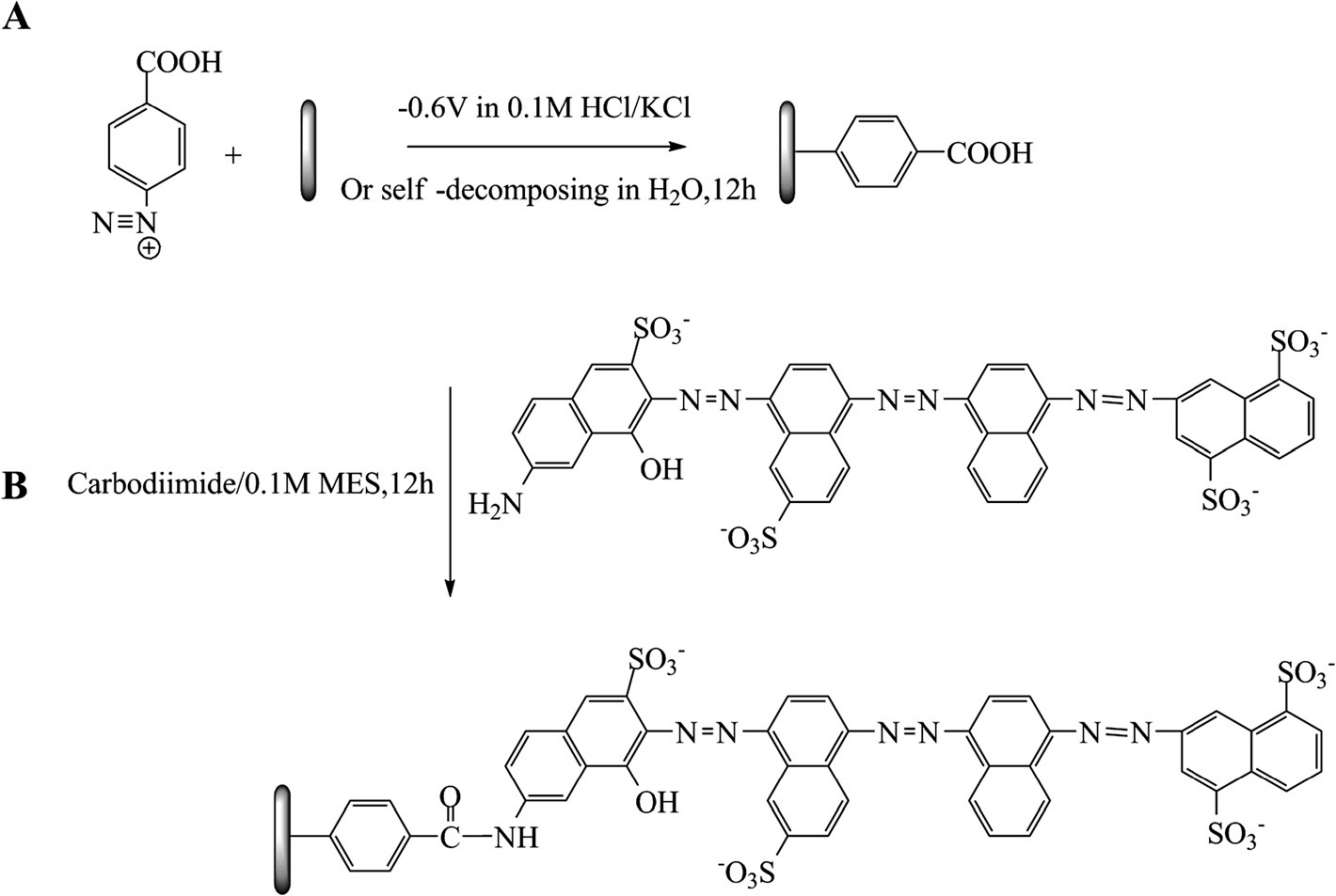
**Schematic illustration of two-step functionalization. (A)** Electrochemical grafting or chemical grafting of 4-carboxyl phenyl diazonium. **(B)** Carbodiimide coupling of Direct Blue 71 dye.

**Figure 3:**
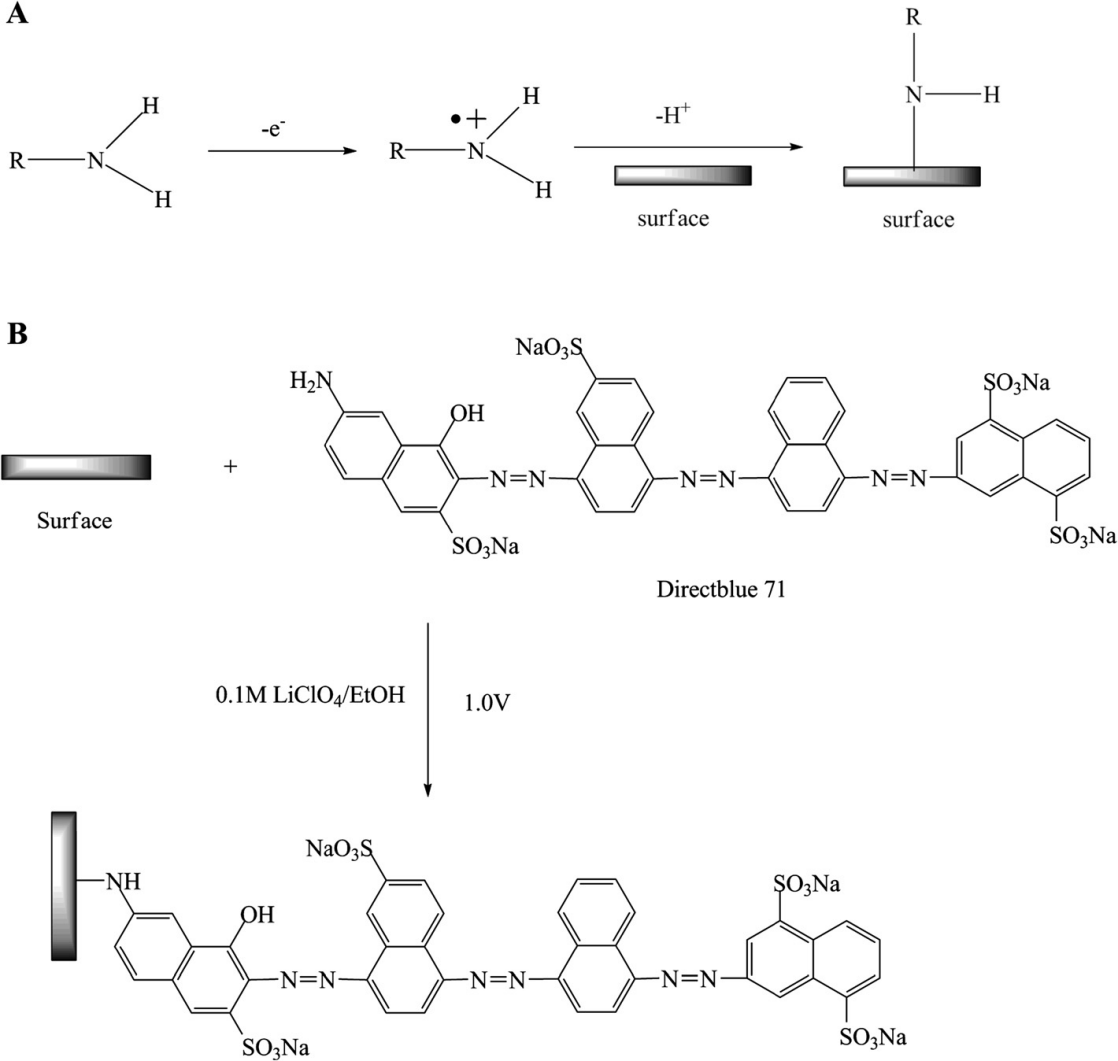
**Schematic mechanism and illustration. (A)** Schematic mechanism of electrochemical oxidation of primary amine on conductive surface. **(B)** Schematic illustration of one-step functionalization of Direct Blue 71 dye via electrooxidation of amine.

The fabrication of DWCNT membranes using microtome cutting method was described in the ‘Methods’ section. TEM image of DWCNTs and SEM image of the as-made DWCNT membrane in cross-sectional view are shown in Figure 1A,B, respectively. Figure 1C shows the schematic structure of functionalized DWCNT membranes with tethered anionic dye. Carbon nanotube membranes can imitate ion channels with the functionalized molecules acting as mimetic gatekeepers. In our previous studies, functionalization of the gatekeeper includes the two-step modification, [18,45] as shown in Figure 2. CNT membranes were first modified by 4-carboxylphenyl diazonium grafting, and then the negatively charged dye molecules were linked with carboxyl sites using carbodiimide coupling chemistry. However, it is difficult to control the gatekeeper density since the oligomer is formed by diazonium grafting and the second coupling reaction may not have 100% yields. The functionalization chemistry at the CNT tip determines the applications for CNT membranes, with the ideal gatekeeper being a monolayer grafted at the entrance of CNT cores that can actively pump chemicals through the pores [13]. The mechanism of electrooxidation of amine includes radical generation and bonding formation on the surface (Figure 3A). The electrooxidation of amine first generates an amino radical cation. After deprotonation, the neutral aminyl radical can be covalently attached to the surface, but the yield is typically less than that of diazonium grafting [46-49]. By electrooxidation of the amine group of dye (as shown in Figure 3B), the charged dye molecules were simply covalently grafted in one-step functionalization.

**Figure 4:**
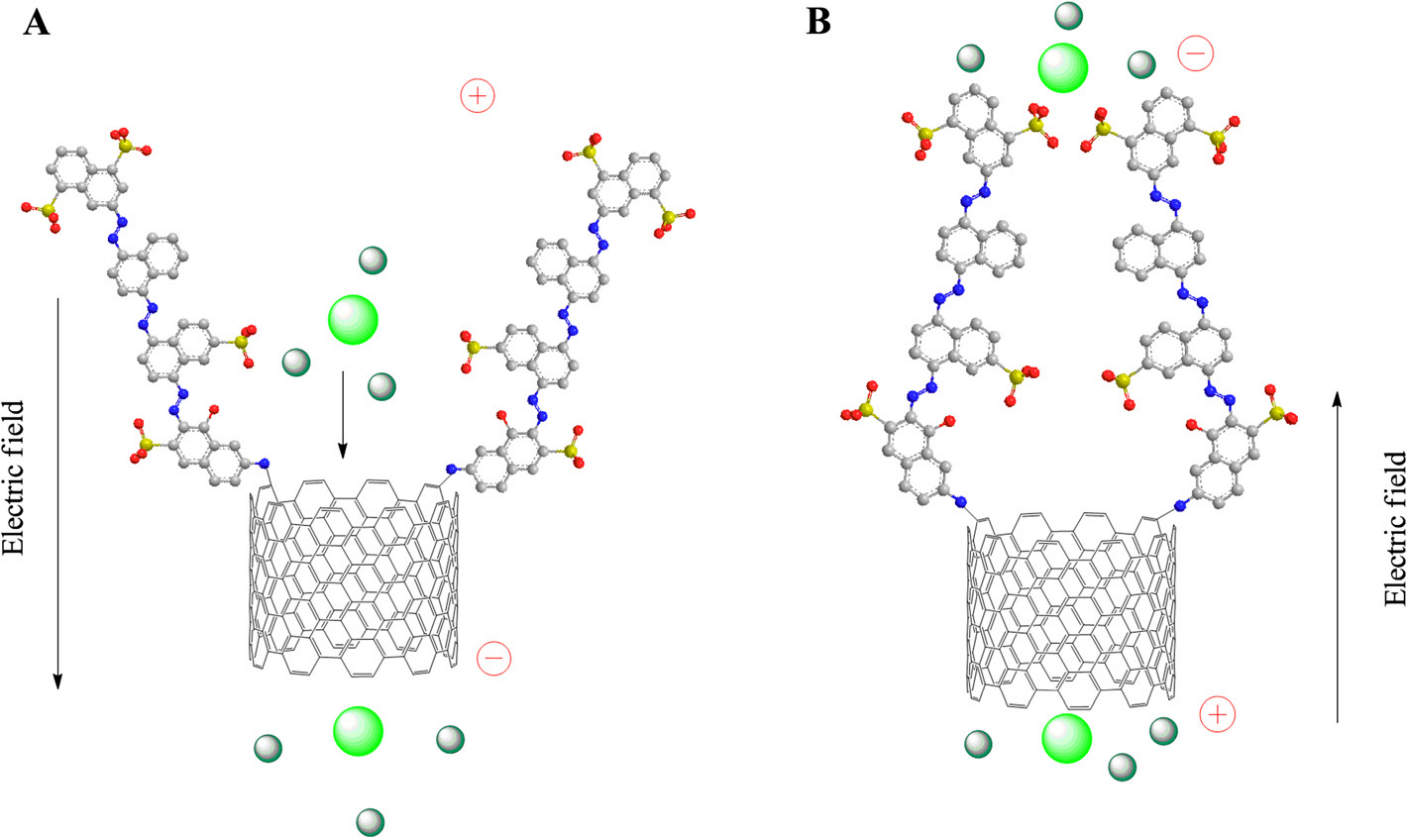
**Schematic mechanism of ionic rectification on DWCNT-dye membrane (A, B).** Gray, C; blue, N; red, O; yellow, S; light green, Fe(CN)_6_^3−^; dark green, K^+^.

In order to compare the gatekeeping efficiency of two different functional chemistries, transmembrane ionic rectification was measured on DWCNT-dye membranes. Figure 4 illustrates the schematic mechanism of ionic rectification on the DWCNT-dye membrane. With a negative applied bias across the membrane, the dye molecules are repelled away from CNT entrance, resulting in an open state, and potassium ions can go through the CNT channel, giving easily measured current. However, at a positive bias, anionic gatekeepers will be dragged into the pore entrance, thus blocking or reducing the ionic current. The rectification experiment setup is diagrammed in Additional file 1: Figure S1. The DWCNT membrane coated with a layer of 30-nm-thick Au/Pd film (working electrode) was placed in U-tube filled with potassium ferricyanide. Ag/AgCl electrode was used as reference/counter electrode. Constant potential was provided using a Princeton Applied Research (Oak Ridge, TN, USA) model 263A potentiostat. Linear scan was ranged from −0.60 to +0.60 V with the scan rate as 50 mV/s. The rectification factor was calculated by the ratio of ionic transport current at ±0.6-V bias.

**Figure 5:**
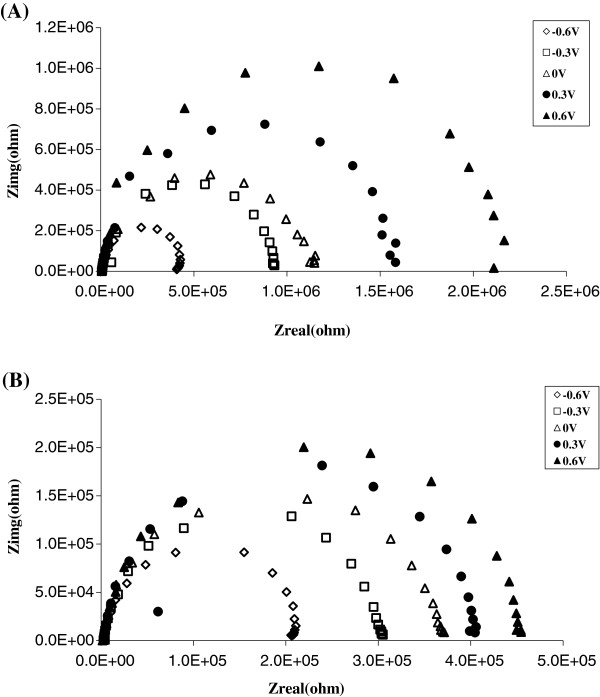
Nyquist plots of dye-modified membrane in (a) 20 mM KCl (b) 100 mM KCl.

Non-faradic EIS measurements were carried out to prove the effectiveness of the one-step electrochemical reaction on DWCNT membranes and demonstrate the conformational changes of tethered dye molecules [42]. The Nyquist plots of EIS are shown in Figure 5A,B, with the frequency ranging from 100 kHz to 0.2 Hz. Platinum wire, Ag/AgCl, and DWCNT-dye membranes were used as counter, reference, and working electrodes, respectively (Additional file 2: Figure S2). By switching the bias from 0 to + 0.6 V, charge transfer resistance was increased (*R*_ct_) 2.3 times in 20 mM KCl (Figure 5A). It indicated that positive bias can draw the negatively charged dye to the CNT entrance, resulting in the blocking of the CNT, reducing ionic current, and increasing *R*_ct_. By applying negative applied bias, *R*_ct_ was reduced two times since the dye molecules can be repelled away from the tip. Under higher concentration at 100 mM KCl, *R*_ct_ was increased only 1.2 times, switching the bias from 0 to + 0.6 V, and a factor of 1.7 times, switching the bias from 0 to −0.6 V (Figure 5B). The slower *R*_ct_ changing rate was due to the ionic screening effect. The results of non-faradic EIS indicated that the gatekeeper can be actuated to mimic the protein channel under bias.

**Figure 6:**
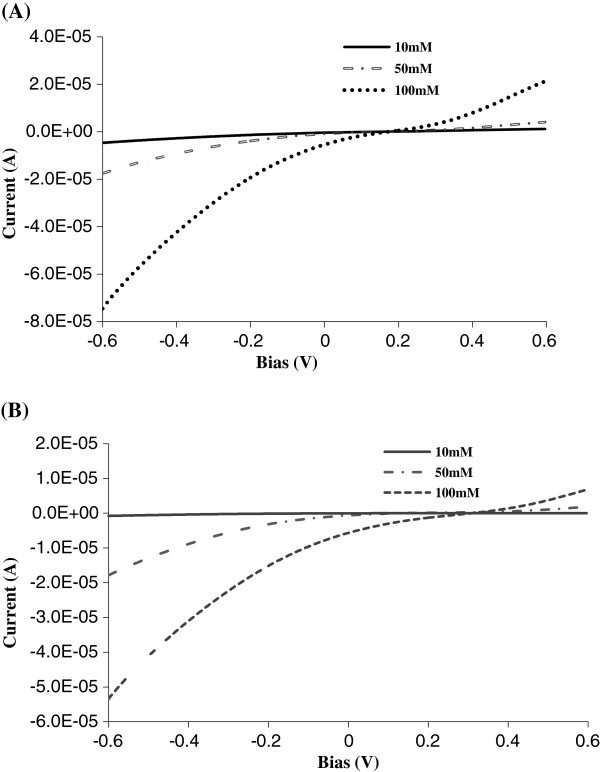
Ionic rectification curves on (A) as-made and (B) modified DWCNT membranes with potassium ferricyanide.

Due to the broad size distribution of double-walled CNT diameter, different membranes varied in initial rectification factors, and comparisons should be made within the same series. Also, due to the relatively large size of DWCNTs (approximately 2.0-nm i.d.) compared to single-walled CNTs (SWCNTs, 1.4 nm), the rectification of small ion pairs (i.e., KCl) was not seen, as was for the case of SWCNTs [42]. However, larger mobile anions such as ferricyanide, 2,6-naphthalenedisulfonic acid (NDS), and benzenesulfonate showed rectification (Table 1). The ionic current of potassium ferricyanide vs. transmembrane bias for as-made and modified DWCNT membranes is shown in Figure 6, with a summary of rectification factors in Table 2. The highest observed experimental rectification factor of ferricyanide was 14.4 for single-step grafting, which was 3.7 times as that of as-made membrane. The rectification factor dropped with increasing ionic concentration, which was expected for the screening of charge on the gatekeepers at high ionic strength. The rectification factor dropped to 9.8 when the ferricyanide concentration increased from 10 to 50 mM. With the concentration increasing up to 100 mM, the rectification factor further dropped to 8.0. It seemed that rectification was attributed to both charge and steric effects at low concentration. The steric effect was dominant at the high-concentration region.

**Table 1:** Summary of ionic rectification factor on single-step modified DWCNT-dye membrane

**Concentration**	**Rectification factor**		
**(mM)**	**Potassium ferricyanide**	**NDS**	**Sodium benzenesulfonate**
10	7.2 ± 0.3	3.1 ± 0.3	2.4 ± 0.2
50	6.4 ± 1	2.0 ± 0.1	2.0 ± 0.1
100	5.6 ± 1	2.3 ± 0.1	1.7 ± 0.1

Rectification factor was calculated by the ratio of ionic transport current at ±0.6-V bias. Linear scan was from −0.60 to +0.60 V with the scan rate at 50 mV/s.

**Table 2:** **Comparison of ionic current rectification factor in K**_**3 **_**Fe(CN)**_**6 **_**solution**

**Concentration of K**_**3**_**Fe (CN)**_**6**_	**Rectification factor**			
**(mM)**	**As-made**	**Single-step electrooxidation of amine**	**Electrochemical grafting of diazonium and coupling of dye**	**Chemical grafting of diazonium and coupling of dye**
10	3.9 ± 0.8	14.4 ± 0.6	2.9 ± 0.2	4.0 ± 0.4
50	4.4 ± 0.9	9.8 ± 0.3	2.9 ± 0.2	3.3 ± 0.07
100	3.4 ± 0.1	8.0 ± 0.4	3.2 ± 0.3	3.6 ± 0.2

Rectification factor was calculated by the ratio of ionic transport current at ±0.6-V bias. Linear scan was from −0.60 to + 0.60 V with the scan rate at 50 mV/s.

On another modified membrane with one-step amine grafting, we compared the rectification factor of three different ions, namely ferricyanide, NDS, and sodium benzenesulfonate, to examine the role of anion size in being repelled by the modification of CNT tips. In Table 1, we saw that as the ion size was reduced, smaller rectification factors were seen, which were consistent with those of partially blocked ion channels. Similar to Table 2, as ionic strength was increased, the rectification factor decreased for all of the anions. It indicated that the rectification was partially attributed to the charge effect. As a control experiment, the single-step grafted dye on DWCNT membranes used in Table 1 was removed by plasma oxidation. As seen in Table 3, the rectification factor dropped to 2 and 3, close to that of the expected as-made membranes. The disappearance of rectification effect provided supportive evidence that the functional anionically charged dye played as gatekeeper to modulate the ionic flux through DWCNT membranes.

**Table 3:** Summary of ionic rectification factor on DWCNT membrane after water plasma oxidation to remove gatekeepers

**Concentration**	**Rectification factor**		
**(mM)**	**Potassium ferricyanide**	**NDS**	**Sodium benzenesulfonate**
10	3.2 ± 0.3	1.7 ± 0.2	2.4 ± 0.2
50	2.8 ± 0.3	1.5 ± 0.07	2.0 ± 0.2
100	2.4 ± 0.2	1.4 ± 0.0.02	2.0 ± 0.2

Ferricyanide has a well-known redox potential of 0.17 V (vs. Ag/AgCl), and thus, an important control experiment was done to make sure that the observed rectification was not due to faradic current; instead, it was due to transmembrane ionic current. Cyclic voltammetry scans (−0.6 to 0.6 V) showed no redox reaction on both as-made and one-step functionalized DWCNT membranes in 50-mM ferricyanide (Additional file 3: Figure S3). We also did not observe redox reaction on glassy carbon in 2-mM ferricyanide, as seen in the flat curve in Additional file 4: Figure S4A. The much larger conductive area of the glassy carbon electrode compared to 5% DWCNT membrane requires the use of more diluted (2 mM) ferricyanide solution. However, with the supporting 0.5-M electrolyte KCl solution, the oxidation and reduction peaks were observed at 0.29 and 0.06 V, which were similar to those found in reports [30,50]. The experiment was also repeated with both redox species. In Additional file 4: Figure S4B, no redox peak was found on glassy carbon in 50-mM ferricyanide solution and 25-mM ferricyanide/ferricyanide solution. The control experiments of cyclic voltammetry on DWCNT membrane and glassy carbon ruled out the redox reaction of ferricyanide, which supports the ionic rectification on electrochemically grafted CNT membranes.

**Figure 7:**
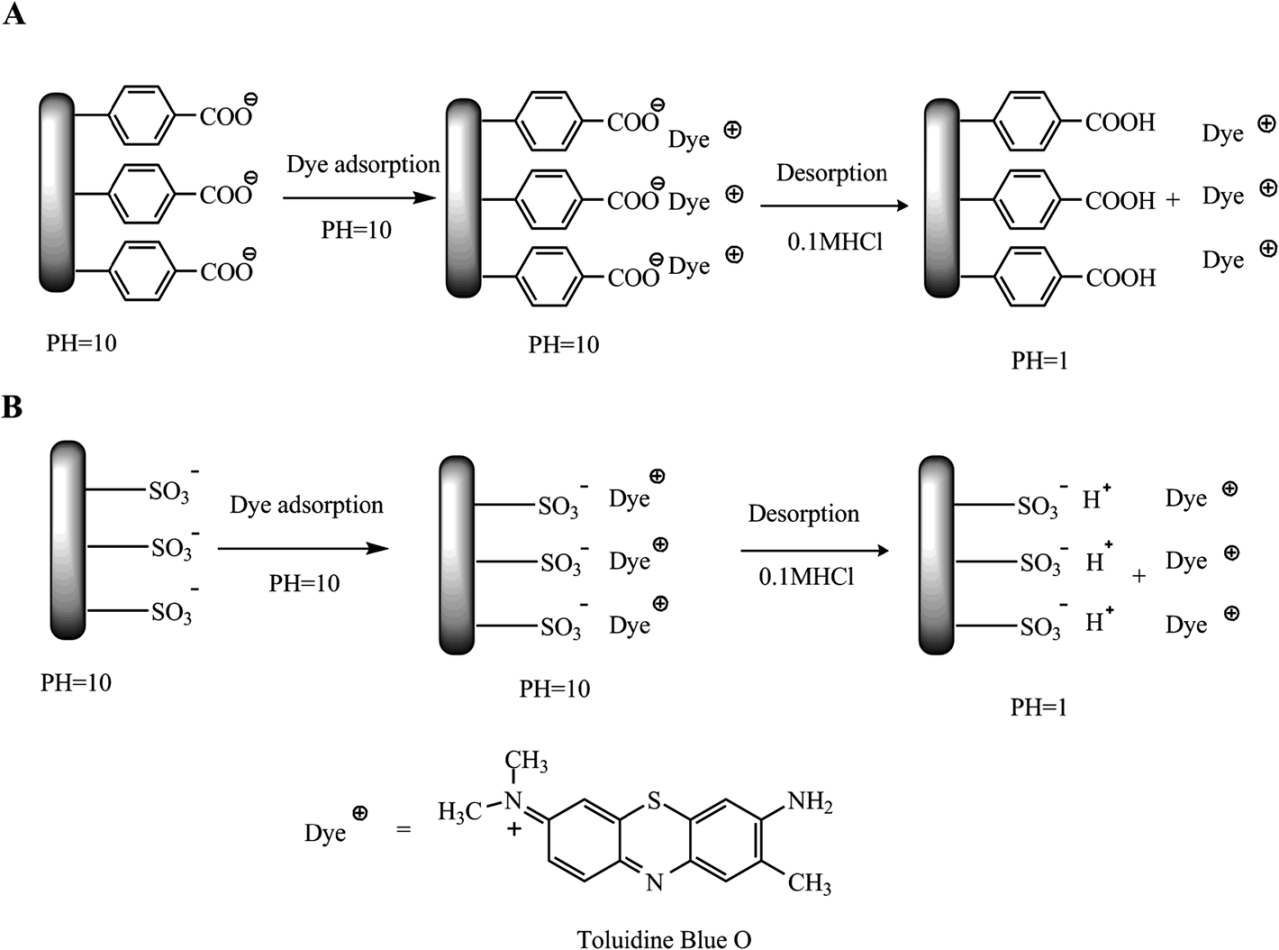
**Schematic illustration of dye assay quantification. (A)** Quantification of carboxylic density on glassy carbon by pH-dependent adsorption/desorption. **(B)** Quantification of sulfonate density by ionic screening effect. (assumed charge/dye = 1:1).

**Figure 8:**
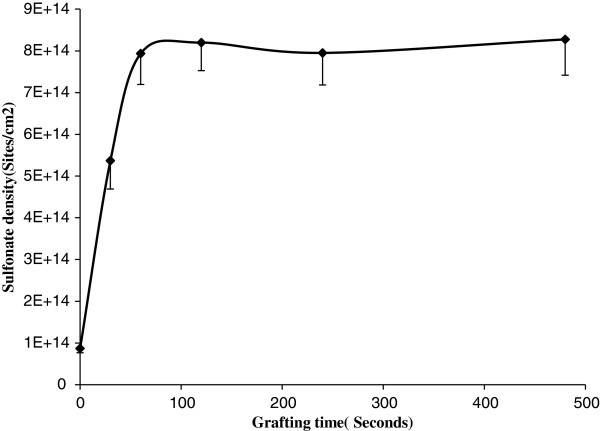
Quantification of sulfonate density as a function of grafting time using dye assay.

The non-faradic (EIS) spectra indicated that the functionalized gatekeeper by a single step can be actuated to mimic the protein channel under bias. This functional chemistry was proven to be highly effective on the enhancement of ion rectification. The disappearance of rectification also supported its effectiveness after removing the grafted gatekeeper by plasma etching. Interestingly, no apparent change of rectification was seen for the two-step functionalization. The likely reason is that highly efficient functional density can be obtained by electrografting of amine in one step since the poor yield in the second step (carbodiimide coupling reaction) resulted in a significantly lower gatekeeper density on CNT membranes. To address this question, two- and one-step functionalizations were quantified using dye assay on glassy carbon due to its well-defined area and similar chemical reactivity to CNTs. The schematic mechanism of dye assay's absorption and desorption is shown in Figure 7. The sulfonate density as a function of one-step amine grafting time is shown in Figure 8. The sulfonate density reached its saturated level at 0.9 × 10^15^ molecules/cm^2^ after 2 min of grafting. Since each Direct Blue 71 dye molecule contains four sulfonate groups, the dye molecule density was calculated as 2 × 10^14^ molecules/cm^2^, nearly one-half of the ideal monolayer density of 3.8 × 10^14^ molecules/cm^2^. The amine grafting density was less efficient than diazonium grafting density, which is consistent with that in the report [49]. Comparison of the total surface charge density by the two grafting methods is shown in Table 4. In the first step of the two-step functionalization, the carboxyl density reached up to 1.3 × 10^15^ molecules/cm^2^ after 8 min of grafting, showing an efficient process. After carbodiimide coupling of dye in the second step, the charged density increased to 2.0 × 10^15^ molecules/cm^2^. With each carboxyl site being replaced with one dye molecule containing four sulfonate groups after coupling, each reacted site will have a net gain of three more charges. Going from 1.3 × 10^15^ to 2.0 × 10^15^ charges/cm^2^, with 3 charges/added dye, resulted in a sulfonate density of 0.93 × 10^15^ charges/cm^2^ after the two-step functionalization. The dye density was calculated as 0.233 × 10^15^ molecules/cm^2^ (one-fourth of the sulfonate density). This resulted in a carbodiimide coupling efficiency of 18% on glassy carbon. The net sulfonate density for the one- and two-step reactions is both comparable at 0.9 × 10^15^ charges/cm^2^, where the less efficient electrochemical oxidation of amine is similar to the loss in efficiency for the carbodiimide coupling reaction. However, in the case of the DWCNT membranes, the two-step modification was not effective at showing rectification (Table 2). There are two possible reasons for the poor rectification on the membrane with two-step modification. The first possible reason is that dye molecules were directly conjugated on the CNT surface via the C-N bond in single-step modification. In two-step modification, the dye molecules were anchored on the diazonium-grafted layer, which is less conductive than glassy carbon. Therefore, the directly grafted dye molecules in a single step are more responsive to the applied electric field. Another possible reason is that the actual yield of the second step in the two-step modification on CNT membranes may be significantly below the 18% yield seen on glassy carbon. The CNT surfaces interfere in the coupling reaction, presumably through the absorption of intermediates.

**Table 4:** Quantification of carboxyl and sulfonate density using dye assay

	**Modification on glassy carbon**	**Charge density**	**Carboxyl density**	**Sulfonate density**
		**(molecules/cm**^**2**^**)**	**(molecules/cm**^**2**^**)**	**(molecules/cm**^**2**^**)**
Step 1 in two-step functionalization	Electrochemical grafting of 4-carboxyl phenyl diazonium for 8 min	1.3 × 10^15^	1.3 × 10^15^	-
Step 2 in two-step functionalization	Carbodiimide coupling of dye	2.0 × 10^15^	1.07 × 10^15^	0.93 × 10^15^
One-step functionalization	Electrochemical grafting of dye by amine oxidation for 8 min	0.9 × 10^15^	-	0.9 × 10^15^

## Conclusions

DWCNT membranes were successfully functionalized with dye for ionic rectification by electrooxidation of amine in a single step. Non-faradic (EIS) spectra indicated that the functionalized gatekeeper by one-step modification can be actuated to mimic the protein channel under bias. This functional chemistry was proven to be highly effective on the enhancement of ion rectification, wherein the highest experimental rectification factor of ferricyanide was up to 14.4. The control experiments supported that the observed rectification was a result of transmembrane ionic current instead of electrochemical reaction of ferricyanide. With the decreasing size of ion, we have observed smaller rectification due to partially blocked ion channels. The rectification was decreased with the higher ionic concentration. It suggested that the rectification is attributed to both charge and steric effects at low concentration, while the steric effect is dominant at high concentration. After removing the dye, the DWCNT-dye membrane exhibited no enhancement of rectification. This control experiment supported that the rectification was induced by functionalized dye molecules. The saturated functionalized dye density by a single step was quantified at 2.25 × 10^14^ molecules/cm^2^ on glassy carbon by dye assay, the same as that of two-step functionalization. However, no apparent change of rectification was observed for two-step functionalization. The dye molecules on the membrane by single-step functionalization are more responsive to the applied bias due to direct grafting on the conductive surface instead of the grafted organic layer. Another possible reason is that the actual yield of the second step of the two-step modification on CNT membranes may be much less than the calculated 18% yield on glassy carbon. One-step functionalization by electrooxidation of amine provides a simple and promising functionalization chemistry for the application of CNT membranes.

## Abbreviations

CNT: Carbon nanotube; EIS: Electrochemical impedance spectra; NDS: 2,6-naphthalenedisulfonic acid.

## Competing interests

The authors declare that they have no competing interests.

## Authors’ contributions

XZ carried out the modification of CNT membranes, rectification measurements and drafted the manuscript. JW fabricated the CNT membranes. ZQC helped in technical support. BH supervised this study and revised the manuscript. All authors read and approve the final manuscript.

## Supplementary Material

Additional file 1: Figure S1 Schematic rectification setup. Working electrode (W.E) is DWCNT membrane coated with 30-nm-thick Pd/Au film; reference/counter electrode (R.E/C.E) is Ag/AgCl electrode. Constant potential was provided using a Princeton Scientific Model 263A potentiostat. Both U-tube sides are filled with potassium ferricyanide (K_3_Fe(CN)_6_) solution. Linear scan from −0.60 to +0.60 V with the scan rate at 50 mV/s. Click here for file

Additional file 2: Figure S2 Schematic setup for the EIS measurements. Experimental conditions: working electrode (W.E), DWCNT-dye membrane; reference electrode (R.E), Ag/AgCl; counter electrode (R.E), Pt; AC magnitude, 10 mV; DC magnitude, −0.6, −0.3, 0, 0.3, 0.6 V; frequency, 100 kHz to 0.2 Hz. Platinum wire, Ag/AgCl, and DWCNT-dye membrane were used as counter, reference, and working electrodes. Click here for file

Additional file 3: Figure S3 Control experiments on DWNT membrane to rule out redox current. Cyclic voltammetry scan on DWNT membrane from −0.6 to +0.6 V. Reference /counter electrode, Ag/AgCl; working electrode, DWNT membrane. Both sides filled with 50-mM potassium ferricyanide solution. No Redox peak is found on bare and modified DWNT membrane, which supports the current change that is from ionic rectification. Click here for file

Additional file 4: Figure S4 Control experiments on glassy carbon to rule out redox current. (A) Cyclic voltammetry scan on glassy carbon in 2-mM ferricyanide solution and 2-mM ferricyanide solution with 0.5 M KCl. (B) Cyclic voltammetry scan on glassy carbon in 50-mM ferricyanide solution and 25-mM ferricyanide/ferricyanide solution (cyclic voltammetry scan from −0.6 to +0.6 V. Reference/counter electrode, Ag/AgCl; working electrode, glassy carbon). With the supporting electrolyte KCl, oxidation and reduction peaks were observed at 0.29 and 0.06 V, respectively. However, no redox peaks were found without KCl, which supports that no redox reaction occurred in the solution. Click here for file
