# Supplementary figures and images for: Single-step electrochemical functionalization of double-walled carbon nanotube (DWCNT) membranes and the demonstration of ionic rectification
Source: Nanoscale Res Lett. 2013 Jun 10;8(1):279. doi: 10.1186/1556-276X-8-279 (PMC3716546; doi:10.1186/1556-276X-8-279)

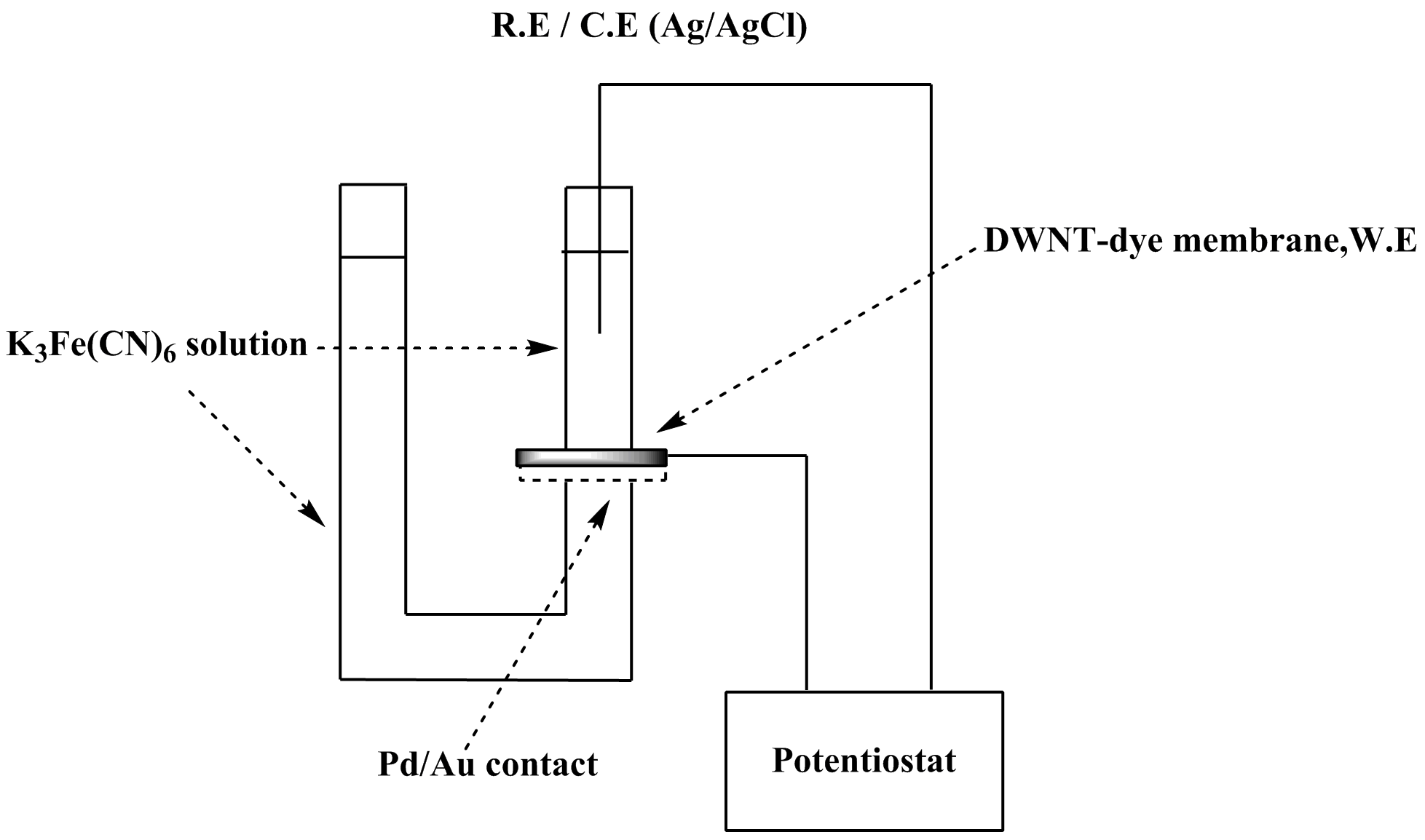

Supplement: Additional file 1: Figure S1 — Schematic rectification setup. Working electrode (W.E) is DWCNT membrane coated with 30-nm-thick Pd/Au film; reference/counter electrode (R.E/C.E) is Ag/AgCl electrode. Constant potential was provided using a Princeton Scientific Model 263A potentiostat. Both U-tube sides are filled with potassium ferricyanide (K3Fe(CN)6) solution. Linear scan from −0.60 to +0.60 V with the scan rate at 50 mV/s. [file 1556-276X-8-279-S1.png]

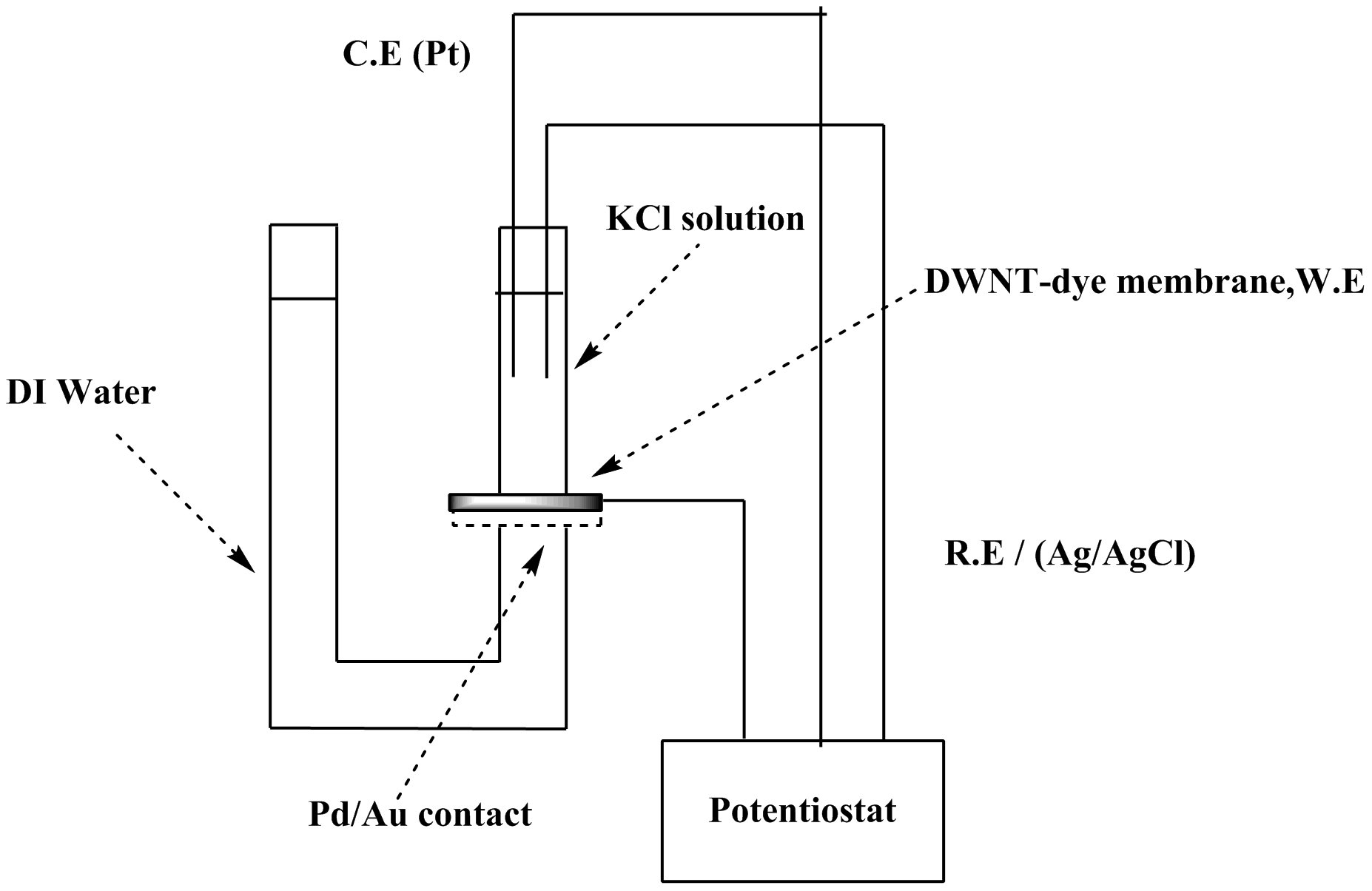

Supplement: Additional file 2: Figure S2 — Schematic setup for the EIS measurements. Experimental conditions: working electrode (W.E), DWCNT-dye membrane; reference electrode (R.E), Ag/AgCl; counter electrode (R.E), Pt; AC magnitude, 10 mV; DC magnitude, −0.6, −0.3, 0, 0.3, 0.6 V; frequency, 100 kHz to 0.2 Hz. Platinum wire, Ag/AgCl, and DWCNT-dye membrane were used as counter, reference, and working electrodes. [file 1556-276X-8-279-S2.png]

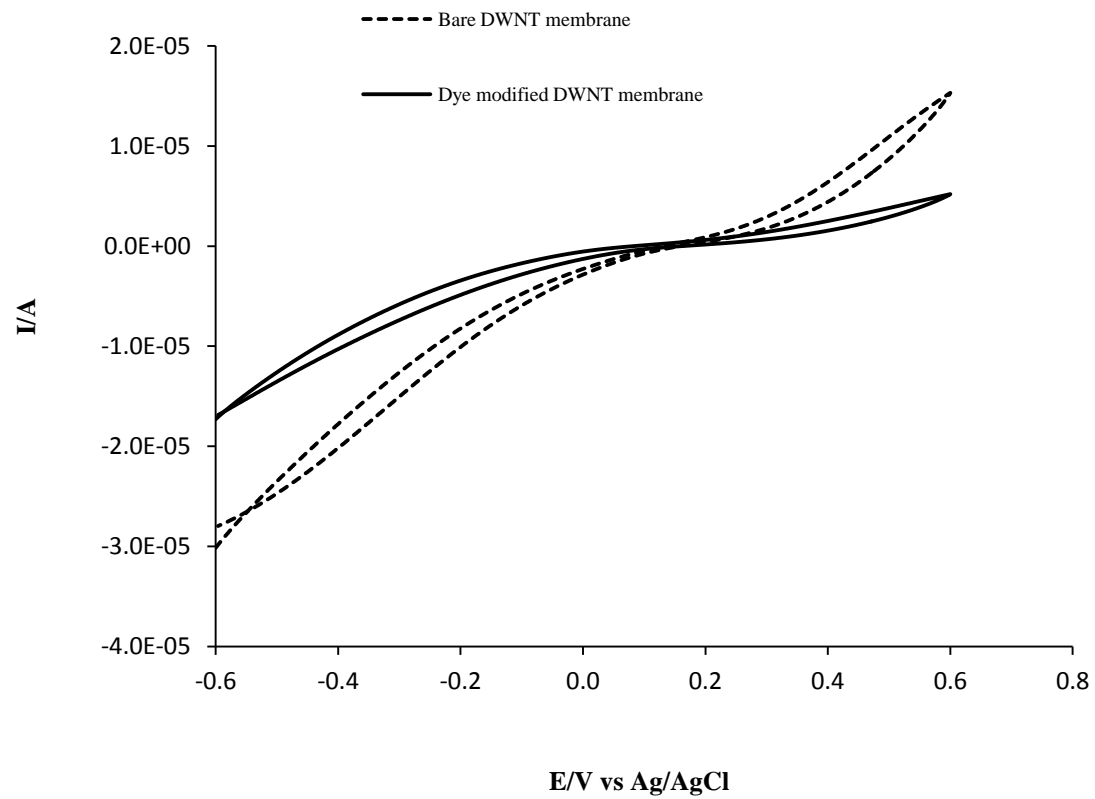

Supplement: Additional file 3: Figure S3 — Control experiments on DWNT membrane to rule out redox current. Cyclic voltammetry scan on DWNT membrane from −0.6 to +0.6 V. Reference /counter electrode, Ag/AgCl; working electrode, DWNT membrane. Both sides filled with 50-mM potassium ferricyanide solution. No Redox peak is found on bare and modified DWNT membrane, which supports the current change that is from ionic rectification. [file 1556-276X-8-279-S3.pdf]

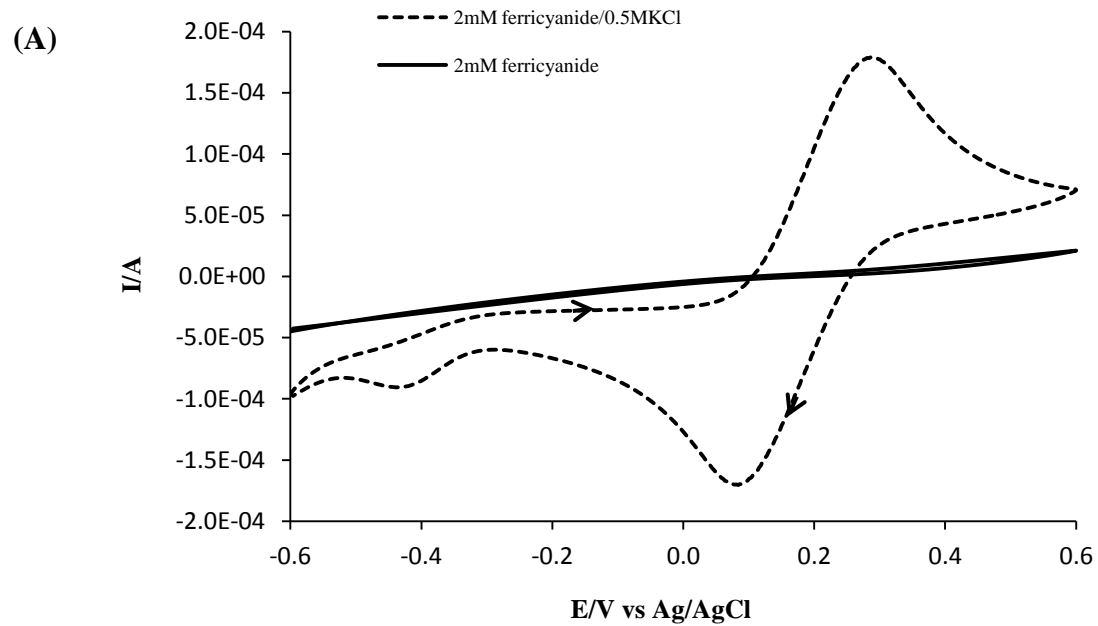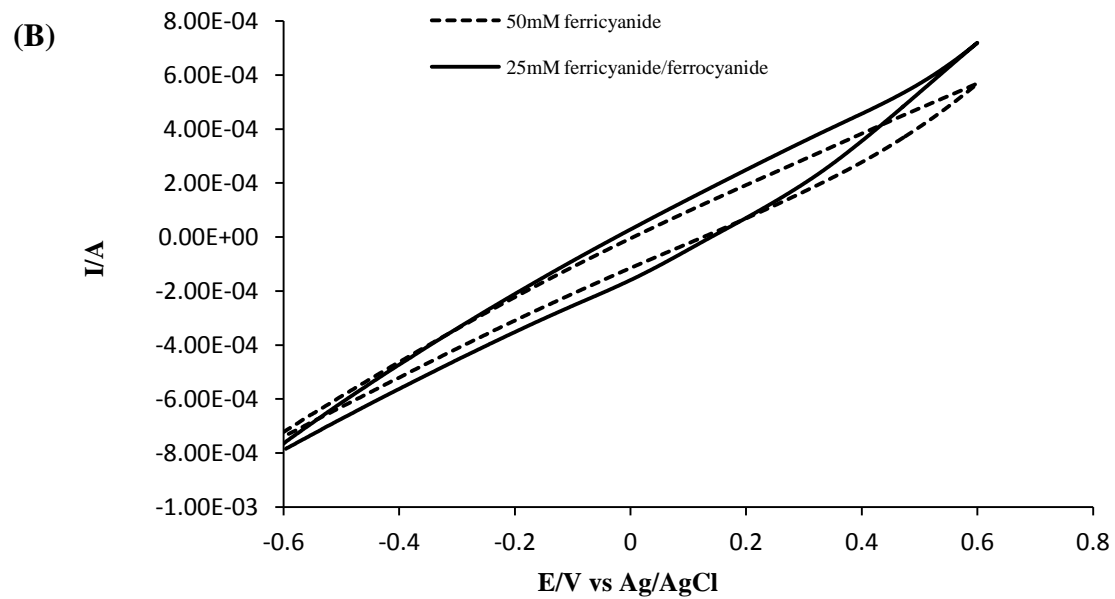

Supplement: Additional file 4: Figure S4 — Control experiments on glassy carbon to rule out redox current. (A) Cyclic voltammetry scan on glassy carbon in 2-mM ferricyanide solution and 2-mM ferricyanide solution with 0.5 M KCl. (B) Cyclic voltammetry scan on glassy carbon in 50-mM ferricyanide solution and 25-mM ferricyanide/ferricyanide solution (cyclic voltammetry scan from −0.6 to +0.6 V. Reference/counter electrode, Ag/AgCl; working electrode, glassy carbon). With the supporting electrolyte KCl, oxidation and reduction peaks were observed at 0.29 and 0.06 V, respectively. However, no redox peaks were found without KCl, which supports that no redox reaction occurred in the solution. [file 1556-276X-8-279-S4.pdf]
